# Beetles as Plant Pathogen Vectors

**DOI:** 10.3389/fpls.2021.748093

**Published:** 2021-10-11

**Authors:** Beata Wielkopolan, Magdalena Jakubowska, Aleksandra Obrępalska-Stęplowska

**Affiliations:** ^1^Department of Monitoring and Signaling of Agrophages, Institute of Plant Protection – National Research Institute, Poznań, Poland; ^2^Department of Molecular Biology and Biotechnology, Institute of Plant Protection – National Research Institute, Poznań, Poland

**Keywords:** beetles, vector, plant pathogens, Coleoptera, viruses, bacteria, fungi, plant diseases

## Abstract

Herbivorous insects, likewise, other organisms, are exposed to diverse communities of microbes from the surrounding environment. Insects and microorganisms associated with them share a range of relationships, including symbiotic and pathogenic. Insects damage plants by feeding on them and delivering plant pathogens to wounded places, from where pathogens spread over the plant. Thus insects can be considered as both pests and reservoirs or vectors of plant pathogens. Although beetles are not mentioned in the first place as plant pathogen vectors, their transmission of pathogens also takes place and affects the ecosystem. Here we present an overview of beetles as vectors of plant pathogens, including viruses, bacteria, fungi, nematodes, and Oomycota, which are responsible for developing plant diseases that can have a significant impact on crop yield and quality.

## Introduction

**Figure 1:**
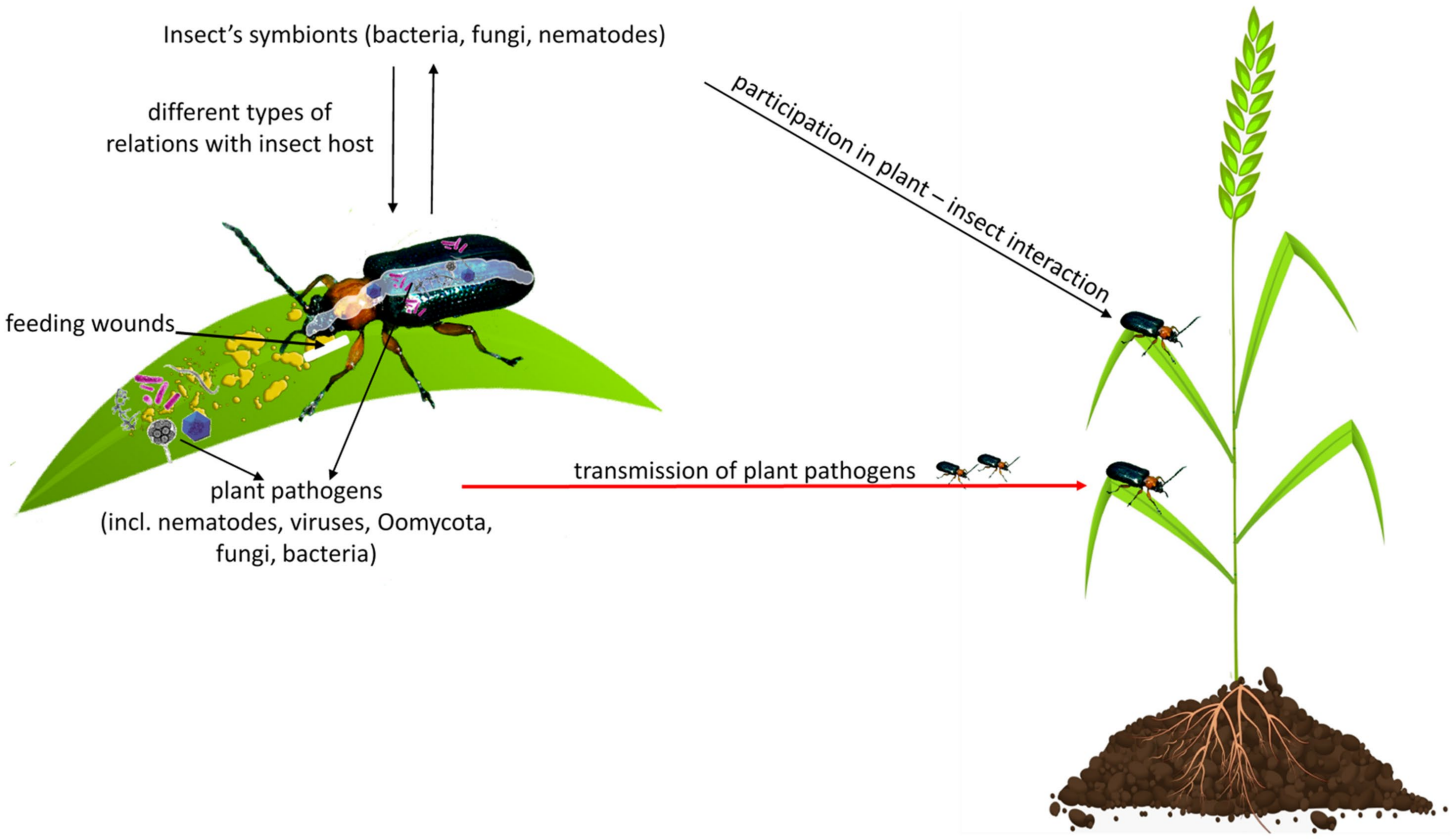
Herbivorous insects are exposed to diverse communities of microbes, including bacteria, fungi, viruses, Oomycota, or nematodes. They share with insects different types of relationships, including symbiotic (mutualism, commensalism, and parasitism) or pathogenic. An insect can damage a plant directly by feeding and indirectly by the transmission of plant pathogens to a wounded place, from where pathogens spread throughout the plant. Insect’s oral secretion or regurgitant may contain microbes that can affect plant response to insect feeding or can be pathogenic for the plant.

Herbivorous insects, likewise, other organisms, are exposed to diverse communities of microbes, including bacteria, fungi, viruses, Oomycota, from the surrounding environment (Hammer et al., 2017; Gurung et al., 2019). Many microbes acquired by insects *via* the diet or soil may not impact insect hosts (Hammer et al., 2017; Zhao et al., 2019). However, some can colonize insects and share with them symbiotic (mutualism, commensalism, and parasitism) or pathogenic relationships. Insects-associated microbes can have diverse roles in mediating insect interactions with plants, other insects, or other microbes (Chung et al., 2013; Mason et al., 2019). It has been shown that insect’s oral secretions or regurgitants contain diverse microbial communities, effectors, proteins, and small molecules that can affect plant defense response to insect feeding (Figure 1; Acevedo et al., 2017; Gedling et al., 2018).

Insects can be considered as both pests and reservoirs or even vectors of plant pathogens because they damage the plant directly by feeding and indirectly by delivering plant pathogens to wounded places, from where pathogens spread throughout the plant (Figure 1; Agrios, 2008).

Hemipteran insects, including leafhoppers and psyllids, are considered by far the most important vectors of bacteria (Perilla-Henao and Casteel, 2016) due to their wide host range and rapid reproduction. In turn, whiteflies and aphids are considered important vectors of viruses (Jones, 2003; Perilla-Henao and Casteel, 2016; Ghosh et al., 2017).

Coleoptera is the largest insect order accounting for over 360,000 species, which constitutes 40% of the known insect species in the world. Beetles are not mentioned in the first place as disease vectors, but some of them cause considerable damages through the transmission of plant pathogens. Here, we present an overview of beetles as vectors of plant pathogens, including viruses, bacteria, nematodes, fungi, and Oomycota, which are responsible for developing plant diseases with a significant impact on crop yield, and quality (Salaau Rojas, 2013; Pan et al., 2018). The successful management of plant diseases requires knowledge on the plant–pathogen–insect vector interactions which is fundamental to reduce the occurrence and spread of the plant diseases, and to limit yield losses as well as the amount of used plant protection chemistry which is very important for the environment.

## Mechanism of Plant Pathogens Transmission

**Figure 2:**
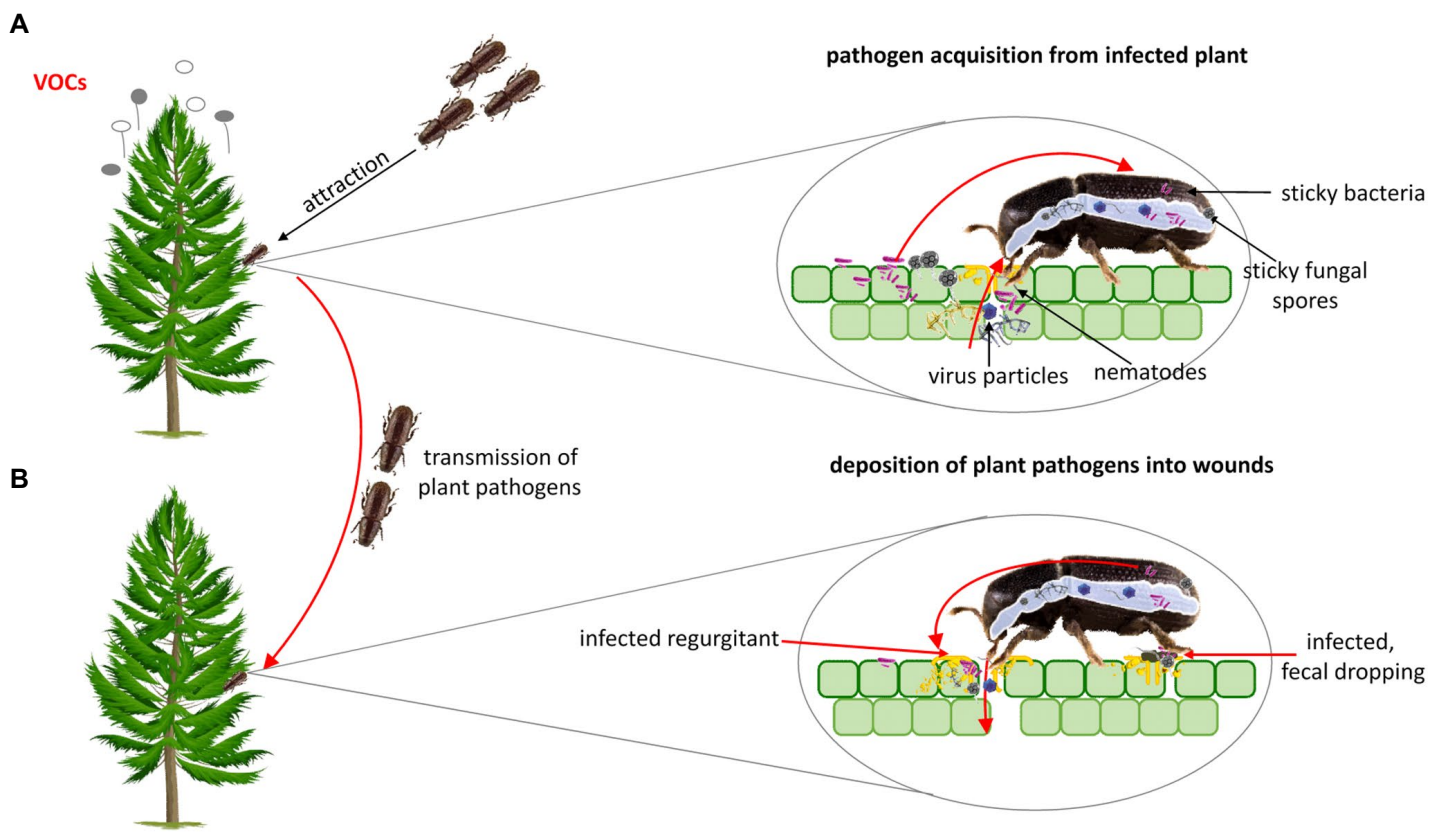
**(A)** Plant pathogens can enhance their acquisition and transmission through altering plant volatile organic compounds (VOCs) that attract insect vector to infected plants. In some cases both the larval and imago stage may be involved in the transmission of plant pathogens. Beetles can acquire plant pathogens incidentally (the bacterial and fungal spores are often sticky and cling to the insect’s body) and with food. **(B)** Ingested pathogen circulates through/within the insect body, reaches the salivary glands, mouthparts, and finally enters plant host through the wounds resulting from insect feeding. Some pathogens can be deposited into wounded place through faecal dropping.

Plants are rooted and motionless, and thus the pathogen must be delivered to them (Agrios, 2008). Natural plant openings or wounds are necessary for the pathogen to penetrate the plant. Insects are considered part of the disease complex because feeding wounds constitute the point of entry for plant pathogens (Willsey et al., 2017). Insects are frequently involved in the transmission of plant pathogens from one plant or organ to another. The way of transmission depends on both, the insect species, and pathogens. In some cases, insects carry pathogens incidentally, without any special relationship between them. For instance, the bacterial and fungal spores are often sticky and cling to the insect’s body during feeding or walking through a plant area where pathogens are deposited. The insect can also acquire the pathogen with food. Ingested pathogen circulates within the insect body, reaches the salivary glands, mouthparts, and finally enters the plant host through the wounds resulting from insect feeding. Overall, insects can carry plant pathogens externally on their legs, mouthparts, bodies, and internally in their digestive tract, and hemocoel (Figure 2; Agrios, 2008; Gedling et al., 2018).

Insects select plant hosts based on the number of sensory cues including visual (e.g., leaf color), olfactory (emission of volatile organic compounds – VOCs; Heard, 1999; Mauck et al., 2014), gustatory, or tactile stimuli (Heard, 1999). Numerous studies suggest that plant pathogens, including viruses, induce changes in plant phenotypes, their palatability, and nutrients components, to enhance visiting of the plants by insect vectors and to increase pathogens acquisition and transmission to other plants (Lieutier et al., 2009; Chesnais et al., 2020). It was reported that beetle vectors have a preference for pathogen-infected plants (Musser et al., 2003). For instance, the induction of changes in the plant VOCs enhances the aggregation of insect vectors on infected plants (Mauck et al., 2010). This phenomenon is observed in many insect species, including these belonging to the Coleoptera order. Pathogens can also affect the quality of the primary plant host as the resource for the insect vector (Mauck et al., 2010; Chesnais et al., 2019) and can have a direct effect on insect behavior (Figure 2; Coplin et al., 2002; Mann et al., 2012; Shapiro et al., 2012). In addition, the increased attraction of insect vectors to infected plant hosts has been documented in response to visual changes in plant phenotype elicited by plant pathogens (Chesnais et al., 2020). Musser et al. (2003) indicated that *Epilachna varivestis* (Coccinellidae) prefers to feed on visually changed plants infected by bean pod mottle virus (BPMV, *Secoviridae*) and southern bean mosaic virus (SBMV, *Solemoviridae*).

## Beetles as Reservoirs and Vectors of Viruses

Several plant viruses are spread by plant contact or their vegetative reproduction, but many of them depend on vectors. More than 70 species of beetles (Smith et al., 2017) are known to transmit viruses that infect economically important vegetables and grain crops. It is estimated that beetles transmit approximately 11% of insect-borne viruses (Smith et al., 2017; Bhat and Rao, 2020). Beetle vectors of plant viruses belong to Chrysomelidae, Coccinellidae, Curculionidae, Meloidae families (Gergerich, 2001; Fereres and Raccah, 2015) and have a unique mode of transmission of at least six groups of plant virus genera: *Machlomovirus*, *Bromovirus*, *Carmovirus*, *Comovirus*, *Sobemovirus*, and *Tymovirus* (Scott and Fulton, 1978; Gedling et al., 2018). Mechanisms of virus acquisition and transmission are associated with the fact that most of the beetle vectors eat plant cells between the leaf veins and regurgitate during feeding, bathing their mouthparts with sap and virus particles, therefore beetle-associated viruses can be deposited into a chewing wound. Virus particles are translocated in the xylem elements to parts of the plants away from the site where they were deposited by an insect (Gergerich and Scott, 1988, 1991; Gedling et al., 2018).

Noncirculative viruses can be transmitted in a semipersistent manner by many groups of insects including beetles (Raccah and Fereres, 2009). These viruses are retained in the foregut, a chitinous anterior region of the insect alimentary canal (Mauck et al., 2018). Some viruses move into the beetle hemolymph immediately after ingestion (Fereres and Raccah, 2015), wherein probably virus is retained for extended periods (Fulton and Scott, 1977; Scott and Fulton, 1978). It was indicated that a beetle could get viruses after a single bite of plant tissue, but the efficiency of acquisition increases with more extensive feeding (Fulton and Scott, 1977). Beetles can acquire and transmit the virus after feeding for a few seconds and can retain the virus from 1 to 10days (Agrios, 2008), depending on the beetle species. For instance, *E. varivestis* retains cowpea severe mosaic virus (CPSMV, *Secoviridae*) for 1day, whereas *Cerotoma trifurcate* (Chrysomelidae) retains the same virus for several days (Fereres and Raccah, 2015), and in turn, *Diabrotica balteata* (Chrysomelidae) vectors bean rugose mosaic virus (BRMV, *Secoviridae*; Table 1) for 3days (Fulton and Scott, 1977).

**Table 1:** Beetle vectors of plant pathogens and their plant hosts.

Plant pathogen (family)	Plant hosts	Insect vector	Insect family
Virus			
Cowpea severe mosaic virus (*Secovirida*)	Soybean	*Epilachna varivestis* (Fulton and Scott, 1974) *Cerotoma trifurcate* (Fereres and Raccah, 2015)	Coccinellidae Chrysomelidae
Southern bean mosaic virus (*Solemoviridae*)	Soybean	*Epilachna varivestis* (Musser et al., 2003)	Coccinellidae
Blackgram mottle virus (*Tombusviridae*)	Soybean	*Epilachna varivestis* (Scott and Phatak, 1979)	Coccinellidae
Cocksfoot mottle virus (Solemoviridae)	Cereals, grass	*Oulema melanopus* (Catherall, 1987) *Oulema galleaciana* (Catherall, 1987)	Chrysomelidae
Bean rugose mosaic virus (*Secoviridae*)	Soybean	*Diabrotica balteata* (Fulton and Scott, 1977) *Cerotoma arcuata* (Fulton and Scott, 1977) *Diabrotica speciosa* (Fulton and Scott, 1977)	Chrysomelidae
Maize mottle mosaic virus (*Tombusviridae*)	Corn	*Oulema melanopus* (Nault, 1978) *Systena frontalis* (Nault, 1978) *Chaetocnema pulicaria* (Nault, 1978) *Diabrotica undecimpunctata* (Nault, 1978) *Diabrotica longicornis* (Nault, 1978) *Diabrotica virgifera virgifera* (Nault, 1978) *Popillia japonica* (Nault, 1978)	Chrysomelidae Scarabaeidae
Bean pod mottle virus (*Secoviridae*)	Soybean	*Cerotoma trifurcata* (Giesler et al., 2002) *Colaspis brunnea* (Giesler et al., 2002) *Colaspis lata* (Giesler et al., 2002) *Diabrotica balteata* (Giesler et al., 2002) *Diabrotica undecimpunctata howardi* (Giesler et al., 2002) *Epilachna varivestis* (Giesler et al., 2002) *Epicauta vittata* (Giesler et al., 2002)	Chrysomelidae Coccinellidae Meloidae
Spindle tuber viroid (*Pospiviroidae*)	Potato	*Epitrix tuberis* (Leach, 1940)	Chrysomelidae
Bacteria			
*Erwinia tracheiphila* (Erwiniaceae)	Cucurbits	*Acalymma vittatum* (Rand and Enlows, 1916) *Diabrotica undecimpunctata* (Rand and Enlows, 1916; EPPO, 1997) *Diabrotica virgifera virgifera* (Toussaint et al., 2013) *Diabrotica barberi* (Toussaint et al., 2013)	Chrysomelidae
*Pantoea stewartii* (Erwiniaceae)	Maize, sweet corn	*Chaetocnema pulicaria* (Esker et al., 2006) *Chaetocnema denticulata* (Agrios, 2008) *Diabrotica undecimpunctata howardi* (Agrios, 2008) *Diabrotica undecimpunctata* (Rand and Enlows, 1916; EPPO, 1997) *Diabrotica longicornis* (EPPO, 1997) *Agriotes mancus* (Agrios, 2008) *Phyllophaga* sp. (Agrios, 2008)	Chrysomelidae Elateridae Scarabeidae
*Ralsonia solanacearum* (Burkholderiaceae)	Potato	*Epitrix tuberis* (Leach, 1940)	Chrysomelidae
*Streptomyces scabiei* (Streptomycetaceae)	Potato	*Epitrix tuberis* (Leach, 1940)	Chrysomelidae
*Pantoea ananatis* (Erwiniaceae)	Cereals, maize	*Oulema melanopus* (Krawczyk et al., 2020) *Diabrotica virgifera virgifera* (Krawczyk et al., 2021)	Chrysomelidae
Fungi			
Blue stain fungi (Ophiostomataceae)	Conifers	*Hylastes macer* (Schowalter, 2018) *Hylastes nigrinus* (Schowalter, 2018) *Steremnius carinatus* (Witcosky et al., 1986) *Pissodes fasciatus* (Witcosky et al., 1986) *Dendroctonus ponderosae* (Amman, 1983) *Ips pini* (Amman, 1983) *Ips acuminatus* (Amman, 1983) *Tomicus* spp. (Pan et al., 2018) *Tomicus yunnanensis* (Lu, 2011) *Tomicus minor* (Lu, 2011) *Tomicus brevipilosus* (Amman, 1983; Lu, 2011)	Curculionidae
	Elm tree	*Scolytus multistriatus* (Faccoli and Battisti, 1997) *Scolytus scolytus* (Webber, 1990) *Scolytus pygmaeus* (Faccoli and Battisti, 1997) *Scolytus triarmatus* (Faccoli and Battisti, 1997) *Hylurgopinus rufipes* (Willsey et al., 2017)	Curculionidae
Oomycota			
*Phytophthora infestans* (Peronosporacea)	Potato	*Epitrix tuberis* (Leach, 1940)	Chrysomelidae
Nematodes			
*Bursaphelenchus xylophilus* (Aphelenchoididae)	Conifers	*Monochamus alternatus* (Mamiya and Enda, 1972) *Monochamus carolinensis* (Linit, 1988) *Monochamus nitens* (Sato et al., 1987) *Monochamus saltuarius* (Sato et al., 1987; Li et al., 2020) *Monochamus marmorator* (Wingfield and Blanchette, 1983) *Monochamus mutator* (Wingfield and Blanchette, 1983) *Monochamus obtusus* (Akbulut and Stamps, 2012) *Monochamus scutellatus* (Bergdahl et al., 1991) *Monochamus titillator* (Luzzi et al., 1984) *Monochamus notatus* (Bergdahl et al., 1991) *Monochamus galloprovincialis* (Akbulut et al., 2008; Pajares et al., 2017; Haran et al., 2018) *Monochamus sutor* (Pajares et al., 2017) *Monochamus urussovi* (Togashi et al., 2008)	Cerambycidae
*Bursaphelenchus cocophilus* (Aphelenchoididae)	Coconut tree	*Rhynchophorus palmarum* (Giblin-Davis et al., 2013)	Curculionidae

Adult beetles and, in some cases, also larvae are very important vectors of plant viruses (Table 1). Nault (1978) found that larvae of *Oulema melanopus* (Chrysomelidae) transmitted maize chlorotic mottle virus (MCMV, *Tombusviridae*) more efficiently in comparison with adults (Nault, 1978). Additionally, *O. melanopus*, as well as *Oulema gallaeciana* (Chrysomelidae), can transmit effectively cocksfoot mottle virus (CfMV, *Solemoviridae*; Catherall, 1987; Table 1) for up to 15days after its acquisition. CfMV is transmitted more efficiently by adults than in the larval stage.

BPMV, a widespread pathogen in the major soybean-growing areas, can be effectively transmitted by several species of the Chrysomelidae family (Table 1; Giesler et al., 2002). BPMV infection can lead to yield reduction and a deterioration in the quality of the soybean seeds. Reduction of crop yield depends on the time of virus infection relative to plant development (Gergerich and Scott, 1996; Gergerich, 1999) and can range between 36 and 52% (Hopkins and Mueller, 1984).

*Epilachna varivestis* is considered to be a severe pest among others of soybean (Fan et al., 1992; Nakamura and Chavez, 2007). Gedling et al. (2018) showed that *E. varivestis* regurgitant is fundamental to the specificity of beetle transmissible viruses. This pest can transmit several plant viruses, including cowpea severe mosaic (CPMV, *Secoviridae*; Fulton and Scott, 1974), SBMV (Musser et al., 2003), or blackgram mottle virus (BMoV, *Tombusviridae*; Scott and Fulton, 1978; Scott and Phatak, 1979; Table 1).

## Bacterial Transmission

Plant bacterial disease can be manifested by several types of symptoms, including blights, galls, and soft rots. Bacteria can be present on the plant surface in droplets and sticky exudates released through cracks, wounds in the infected area, or through natural openings (including stomata, nectar rhodes, and hydathodes). Insects can be attracted by sweet bacterial exudates. During insect feeding, bacteria stick to mouthparts and other parts of the insect body (Agrios, 2008). Some bacteria obtained with plant material migrate to the insect gut epithelium and are deposited on wounds through infected fecal droppings (Mitchell and Hanks, 2009; Figure 2). For the development of a new bacterial infection, a fresh wound or the natural opening and enough moisture in the plant surface are needed. Thanks to this, bacteria multiply and move into the plant, and bacterial infection is developing (Agrios, 2008).

There are much more currently known and described beetles as vectors of viruses and fungal pathogens than beetles that transmit bacterial pathogens. However, metagenomic studies, including microbiome analyses, may soon provide a lot of valuable information about beetles as reservoirs or even vectors of other plant pathogenic bacteria.

### Bacterial Wilt of Cucurbits

*Erwinia tracheiphila* (Erwiniaceae) causes wilt of cucurbits (Rojas et al., 2011), which can be responsible for millions of dollars yield losses and additional costs spent on indirect preventative measures (Shapiro et al., 2014). Bacterial wilt is dangerous for many cucurbit crops, causing losses of up to 80% (Rojas et al., 2011). *Erwinia tracheiphila* is unable to infect the cucurbits through the natural openings of the plants such as stomates or hydathodes, thus wounding, including those caused by insects is needed for pathogen entry and developing the disease (Ferreira and Boley, 1992). Two species of the Chrysomelidae family: *Acalymma vittatum* and *Diabrotica undecimpunctata* (Stephenson et al., 2004; Du et al., 2008; Sasu et al., 2010) are involved in the spreading of *E. tracheiphila* (Table 1). Toussaint et al. (2013) indicated that *Diabrotica virgifera virgifera* and *Diabrotica barberi* (Chrysomelidae) might also be involved in the transmission of this pathogen (Table 1). Beetles acquire this bacterium during feeding on infected cucurbit plants. *Erwinia tracheiphila* migrates to the insect gut epithelium (Mitchell, 2004) and is deposited at sites of foliar feeding damage on healthy leaves through infected fecal droppings (Yao et al., 1996; Mitchell and Hanks, 2009). Bacteria migrate toward wounds when an aqueous film on the leaf surface is sufficient (Ferreira and Boley, 1992), next multiply in xylem vessels, where excrete polysaccharides, secrete enzymes that break down some of the cell wall substrates and induce xylem parenchyma cells to produce tyloses (outgrowths/extragrouth on parenchyma cells of xylem vessels that can fall from the cells during plant stress or infection). As a result, gels and gums are formed that block vessels and reduce the upward flow of water in the xylem by up to 80%. Finally, the leaves and vines wilt (Agrios, 2008). Shapiro et al. (2012) reported that *E. tracheiphila* alters the foliar and floral VOC emission of its plant host (*Cucurbita pepo* var. *texana*) in comparison to healthy plants. In this way, changes in plant VOCs lead to enhancement of aggregation of insect vectors on infected plants and subsequent pathogen transmission to other plants.

### Bacterial Wilt of Maize: Stewart’s Bacterial Wilt

*Pantoea stewartii* (Erwiniaceae) causes Stewart’s vascular wilt and leaf blight of maize and sweet corn (Coplin et al., 2002), which is responsible for serious crop losses throughout the world (Coplin et al., 2002). This bacterium is unable to spread from plant to plant without an insect vector (Menelas et al., 2006). Several beetle species of the Chrysomelidae, Elateridae, and Scarabeidae families are vectors of *P. stewartii* (Table 1). In the United States, the spreading of Stewart’s wilt disease is fundamentally associated with the *Chaetocnema pulicaria* (Chrysomelidae; Esker et al., 2006). More precisely, the incidence of the disease depends on the winter weather conditions affecting the *C. pulicaria* population because the severity of the disease depends on the number of insects that have survived winter (Nadarasah and Stavrinides, 2011; Bae et al., 2015). Beetles acquire bacterium during feeding on infected corn plants, harbour bacteria along the alimentary tract (foregut, midgut, hindgut; Menelas et al., 2006; Nadarasah and Stavrinides, 2011; Orlovskis et al., 2015), where bacteria remain for the entire duration of the insect’s life (Nadarasah and Stavrinides, 2011). After overwintering time, beetles exit their dormancy stage and start feeding, during which they transmit the bacteria into the feeding wounds *via* their feces (Esker and Nutter, 2002; Menelas et al., 2006). As a result, bacteria enter the vascular tissue of corn leaves and cause disease development (Nadarasah and Stavrinides, 2011).

### Pantoea ananatis

*Pantoea ananatis* (Erwiniaceae) can be associated with plants as an epiphyte, endophyte, pathogen, or symbiont (Lodewyckx et al., 2002; Coutinho and Venter, 2009). That bacterium can cause disease symptoms in a wide range of economically important crops (including in *Cattleya* sp., *Musa* sp., *Cassia pectuta*, sugarcane) or forests (Coutinho and Venter, 2009). For instance, losses of up to 100% were recorded in the cultivation of onions (Gitaitis and Gay, 1997). New reports of disease occurring on a yet unrecorded host are noted. It was established that bacteria enter plants through flowers (Hasegawa et al., 2003; McLeod et al., 2005), wounding caused by insect feeding (Wells et al., 2002; Gitaitis et al., 2003; McLeod et al., 2005; Coutinho and Venter, 2009), mechanical damages (Serrano, 1928), and plant-to-plant contact (Cother et al., 2004). The transmission of *P. ananatis* by insects is relatively unknown. Gitaitis et al. (2003) connected disease symptoms on onion with tobacco thrips vector. In the case of beetles, Krawczyk et al. (2020) indicated that *P. ananatis* isolated from *O. melanopus* was able to develop disease symptoms on wheat plants. It was also reported that *D. virgifera virgifera* is associated with *P. ananatis* (Krawczyk et al., 2021). Obtained results suggest that both beetle species can act as potential reservoirs or vectors of this pathogen.

## Fungal Transmission

The transmission of fungi by insects occurs usually accidentally. Insects can be contaminated with the fungus or its spores during visiting infected plants, externally (for instance during walking) or internally (through feeding). Spores and mycelia adhering to insect bodies or ingested by insects are transported to healthy plant tissues (Willsey et al., 2017; Figure 2). Some of the beetles have special organs, namely mycangia, for carrying fungi (Amman, 1983).

### Blue Stain Fungi

Blue stain fungi (Ophiostomataceae) are necrotrophic pathogens that are associated with various conifers and bark beetle species of the Curculionidae family (Table 1; Amman, 1983; Pan et al., 2018). Beetles vector blue stain fungi of varying virulence that penetrate the tree tissue when the insects tunnel in the phloem (Zhou et al., 2002; Masuya et al., 2003). Fungi can colonize phloem and xylem tissue away from the bark beetle tunnels, capturing tree resources (Six, 2012). The tree dies due to the girdling of both insect adults and larvae and blockage of the tree’s conductive vessels by the fungus (Amman, 1983).

Interestingly, blue stain fungi can elicit tree defenses (Viiri et al., 2001; Lieutier et al., 2009; Shapiro et al., 2012) likely to the benefit of their insect host (Paine et al., 1997; Pan et al., 2018) and can be involved in the production of plant pheromones attracting the insect vectors (Zhao et al., 2015).

The economic losses caused by beetles in combination with blue stain fungi can be huge. For instance, pine shoot beetle from the genus *Tomicus* (*T. yunnanensis*, *T. minor*, *T. brevipilosus*; Table 1) destroyed 93,000ha of economically and ecologically important conifers of Yunnan pine (*Pinus yunnanensis*) in Southwest China since the 1980s (Lu, 2011; Pan et al., 2018).

*Leptographium wageneri* can be responsible for black-stain root disease (BSRD), which can cause considerable damages in conifers forests, for instance, in Northwest America. BSRD can lead to growth reduction, chlorosis development, dark staining of the tracheids from the roots to the lower bole, and ultimately tree death (Jacobs and Wingfield, 2001). Insect vectors play a major role in the spreading of *L. wageneri* inoculums (Table 1). Fungal conidia are produced in sticky masses (conidial droplets) at the apex of stalked conidiophores, inside the galleries created by the bark beetle. Two pests of the Curculionidae family *Hylastes macer* and *Hylastes nigrinus* (Table 1), are the most important vectors of *L. wageneri* in Douglas-fir and ponderosa pine plantations in the western United States (Schowalter, 2018). In the pines tree, *L. wageneri* is spread by *H. nigrinus* and by several long-snouted weevils of the Curculionidae family: e.g., *Steremnius carinatus*, *Pissodes fasciatus* (Curculionidae; Table 1).

The fungus *Ophiostoma ulmi* and *Ophiostoma novo-ulmi* cause vascular wilt disease of elm trees (Dutch elm disease, DED; Brasier, 1991), which is considered one of the most destructive diseases of the woody tree. It was estimated that *O. ulmi* destroyed approximately 10% of the European elm population (Brasier, 2001). Above mentioned fungi naturally spread to new hosts *via* root grafts, but its insect vector transmission is the most important way of dispersal (Table 1). *O. ulmi* and *O. novo–ulmi* are in a close association with the bark beetles from the genera *Scolytus* and *Hylurgopinus* (McLeod et al., 2005; Willsey et al., 2017; Table 1), that spread fungi over large areas. It has been suggested that more than 99% of the elm tree infection are caused by the fungus transmitted by the elm bark beetle. Insect adults can carry on their bodies thousands of fungal spores, which are deposited in the wounded moist tissues of the tree. It is worth mentioning that trees infected by fungus responsible for DED cause higher production of sociochemicals, which attract the insect vector *Hylurgopinus rufipes* (Curculionidae), which increase the efficiency of spreading this pathogen (McLeod et al., 2005; Willsey et al., 2017).

## Oomycota

*Epitrix* sp. (Chrysomelidae) are considered a serious pest of various species of plants. For instance, *Epitrix tuberis* is primarily associated with members of Solanaceae family, especially with potato plants (Malumphy et al., 2016). Both adults and larvae of *E. tuberis* are harmful to plants. It was noted that *E. tuberis* may enhance dispersal of the *Phytophthora infestans* (Peronosporacea; Leach, 1940; Table 1), causing the potato blight. Interestingly, *E. tuberis* can act as a multivector. The role of this insect in spreading various plant pathogens was reported in the past in the literature. It was indicated that *E. tuberis* can transmit bacteria *Raltsonia solanacearum* (Burkholderiaceae) responsible for potato brown rot, *Streptomyces scabiei* (Streptomycetaceae) causing potato scab, and potato spindle tuber viroid (PSTV, Pospiviroidae; Table 1; Leach, 1940). Generally, all species of *Epitrix* sp. can transmit plant pathogens that may have a negative impact on crop yield.

## Nematodes Transmission

Transmission of nematodes can take several ways including direct contact between plant roots, through contaminated tools, or insect vectors. Generally, nematodes transmitted by beetles migrate to trees through wounds caused by beetle feeding and through the oviposition slits in the bark (Agrios, 2008). Healthy plants may also become contaminated by nematodes through insect feces (Bierhals et al., 2018).

### Pine Wilt

Pine wood nematode (PWN, *Bursaphelenchus xylophilus*, Aphelenchoididae) is an invasive pathogen that causes pine wilt disease (PWD). Significant losses of pines caused by PWD were reported, e.g., in Japan, Korea, China, and Portugal (Cheng et al., 2013; Alves et al., 2016; Van Nguyen et al., 2017). For instance, the damaged area of PWD covered 7,829ha in Korea, in 2008 (Han et al., 2008). It is noted that 21 species of Cerambycidae, one species of Budrestidae, and two species of Curculionidae are related to PWD worldwide (Li et al., 2020). But species of the *Monochamus* genus (Cerambycidae; Table 1) are considered the principal vectors of PWD (Filipiak et al., 2021). The nematode special fourth-stage dispersal juveniles are adapted to survive in the respiratory system (trachea) of beetle vectors. Nematodes enter the beetle’s tracheal system *via* openings in the beetle’s exoskeleton (spiracles). Nematodes are transmitted by beetles and enter the tree through the wounds caused by beetle feeding or oviposition slits in the bark. Next, adult nematodes are produced that migrate from the cambium to the resin canals, xylem, and cortex (Agrios, 2008). PWD contributes to plant death by blocking water conductance through the xylem. The damaged tree is visited by females of beetle that lay eggs. *Monochamus* larvae develop in the tree cambium and borrow into the wood. When new beetles emerge, the PWDs migrate to the insect respiratory system (Mota and Vieira, 2008; Mota et al., 2009).

### Red Ring of Coconut Palms

Red ring disease (RRD) is a highly lethal disease (Bierhals et al., 2018) caused by red ring nematode (RRN; *Bursaphelenchus cocophilus*, Aphelenchoididae), which invades parenchymal tissue in the roots, stems, leaves, and artificially infested nuts. The most characteristic infection symptom is an orange to brick-red colored ring in a cross-section of the stem. RRN causes the development of tyloses in xylem vessels blocking the upward movement of water and nutrients (Bierhals et al., 2018). Consequently, leaves become short and deformed. They wilt and die after turning color from yellow bronze to deep reddish-brown (Esser and Meredith, 1987). Due the RRD around 35% of young coconut trees in Trinidad and 80% of trees of coconut trees of one plantation in nearby Tobago died (Esser and Meredith, 1987). Transmission of nematodes to other plants can take several ways, through direct contact between infected and healthy roots, contaminated tools, or insect vectors (Bierhals et al., 2018). Red palm weevil *Rhynchophorus palmarum* (Curculionidae) is a host and a vector of RRN (Table 1). It is estimated that 72% of *R. palmarum* population is associated with RRD (Esser and Meredith, 1987). Cut palm leaves exude compounds that attract *R. palmarum*. Healthy plants are primarily contaminated by nematodes through insect feces or female oviposition on the plant leaf axils (Bierhals et al., 2018). Next, nematodes penetrate the plant tissues through wounds caused by insect feeding (Bierhals et al., 2018). Larvae of weevils are being inoculated by nematodes during feeding on infected red ring tissue. RRN enters the hemocoel of weevil larvae *via* the gut track. In adult weevils, this nematode can be in the gut, body cavity, and ovipositor region. Infected adult weevils emerging from trees can transmit the invasive third-stage larval nematodes ready to infest a new tree (Agrios, 2008).

## Consequences of Pathogen Transmission by Beetles

The transmission of plant pathogens can affect all the components of the pathogen – plant – beetle vector system. The components of this system are additionally affected by environmental factors and the plant protection strategies aimed to reduce the spread of pathogens and pests. In general, the consequences of these three-way interactions are manifold. The examples are listed below.

For the plant, both beetle feeding and damages caused by pathogens are harmful and can have a negative impact on the crop quantity and quality as well as plant growth and development. Sometimes damages may result in the death of the plant. Importantly, in this situation, the plant has to deal with two stressors (pathogens and beetles) at the same time, and fine-tune its defence response at the lowest possible cost of energy (Wielkopolan and Obrępalska-Stęplowska, 2016).

The microbes influence many aspects of insect host life, including adaptation to new environmental niches or plant hosts (Henry et al., 2021), which is beneficial for both insects and microorganisms. From the ecological point of view, the plant pathogens dispersal by vectors is a key factor of distribution and incidence of some plant diseases. Insect vectors may benefit from insect-associated microorganisms (Musser et al., 2003) because microbes can modulate plant defence mechanisms in favour of their insect vector (Paine et al., 1997; Wielkopolan and Obrępalska-Stęplowska, 2016; Pan et al., 2018). It was shown that insect-associated bacteria can cause that the plant defence against insects is milder. For instance, it was indicated that *O. melanopus*-associated bacteria suppressed the expression of wheat genes encoding of harmful to insects serine protease inhibitors (Wielkopolan et al., 2018). This situation might also apply to pathogens.

Vector-borne pathogens can also alter the phenotype of the plant, including its palatability and quality to enhance the aggregation of insect vectors on the infected plant (Mauck et al., 2010). For instance, larvae of *E. varivestis* grow faster on virus-infected leaf tissue. This suggests that the virus – *E. varivestis* relationship might be potentially beneficial for insects since larger insects typically have a higher reproductive potential and they are more likely to escape natural enemies, which increases the chances of transmitting pathogens (Musser et al., 2003).

## Conclusion

Beetles with chewing mouthparts disrupt tissue continuity during feeding and wounding caused by them can constitute a point of entry to plant pathogens. This article presented examples and mechanisms of phytopathogen transmission by beetles. For a plant disease to initiate and develop, the common host for pest and pathogen is required as well as the synchronization between the plant development and the appearance of beetle vector and the pathogen. Climate changes may affect the spread of new alien species of beetle and pathogens to new areas, as a result, new trophic relationships between them can be established. There are still significant gaps in our understanding of beetle-plant-pathogen interactions and their consequence for disease incidence and pathogen spread. Therefore, the main challenges for future research are to understand the mechanism of (a) the acquisition and transmission of the plant pathogens by the insect vectors, (b) plant defense response against insects and associated with them pathogens, and (c) the impact of the pathogen on its insect vector. Research at the molecular level and metagenomic studies may provide a lot of valuable information about three-trophic interactions. Obtained knowledge should provide a more holistic understanding of disease dynamics and will allow for guiding effective monitoring and developing effective tools to limit pathogen transmission and disease incidence.

## Author Contributions

AO-S and BW: conceptualization, literature review, data analysis, and writing – original draft preparation. BW, MJ, and AO-S: writing – review and editing. AO-S: funding acquisition. All authors contributed to the article and approved the submitted version.

## Funding

This work was supported by the Polish National Science Centre within the UMO-2016/23/B/NZ9/03503 agreement.

## Conflict of Interest

The authors declare that the research was conducted in the absence of any commercial or financial relationships that could be construed as a potential conflict of interest.

## Publisher’s Note

All claims expressed in this article are solely those of the authors and do not necessarily represent those of their affiliated organizations, or those of the publisher, the editors and the reviewers. Any product that may be evaluated in this article, or claim that may be made by its manufacturer, is not guaranteed or endorsed by the publisher.
